# A Non-Destructive Moisture Detection System for Unshelled Green Tea Seed Kernels Based on Microwave Technology with Multi-Frequency Scanning Signals

**DOI:** 10.3390/s25051324

**Published:** 2025-02-21

**Authors:** Bo Zhou, Ye Yuan, Zhenbo Wei, Siying Li

**Affiliations:** 1 Department of Mechanical Engineering, Yancheng Institute of Technology, Yancheng 224051, China; zjzhobo@163.com (B.Z.); yuanye1634@163.com (Y.Y.); 2 Department of Biosystems Engineering, Zhejiang University, 866 Yuhangtang Road, Hangzhou 310058, China; weizhb@zju.edu.cn

**Keywords:** unshelled tea seeds, moisture prediction, frequency optimization, attenuation spectrum, artificial neural network

## Abstract

A self-developed microwave moisture detection system (ranged from 2.00 GHz to 10.00 GHz) based on multi-frequency sweep technology was used to quickly determine the moisture content of tea seed kernels without breaking the shells. A multi-frequency evaluation method combined cross-validation and majority voting rules was proposed to select the optimal microwave features from the original microwave signals. Firstly, the moisture content of tea seed kernels was detected by the moisture detection system, and the determination coefficients of the ANN model established based on seven attenuations and six phase-shifts were over 0.999. Then, the moisture content of unshelled tea seeds was detected, and the determination coefficients of the ANN model based on 13 preferred frequency features were over 0.995. Moreover, the predicted moisture values of unshelled tea seeds were calibrated accurately by a moisture function (y = −0.017x^2^ + 1.431x − 1.019). Above all, the self-developed system could achieve non-destructive moisture content prediction of tea seed kernels.

## 1. Introduction

Green tea, scientifically known as *Camellia sinensis*, is one of the most widely consumed beverages globally, renowned for its health benefits and cultural significance. In China, as the birthplace of tea, it is not only a vital agricultural product but also deeply embedded in the nation’s cultural and economic fabric. Tea cultivation supports millions of farmers and contributes significantly to rural economies, In 2023, China’s tea market reached CNY 3347 billion, with projections to grow to CNY 4276 billion by 2028.

Green tea seed oil, as a healthy vegetable oil recommended by Food and Drug Administration, is processed by pressing the seed kernels, which is rich in a variety of unsaturated fatty acids and natural active ingredients (such as tea polyphenols and plant sterols) [1,2,3]. From tea-tree fruit to the tea seed kernel suitable for oil extraction, the pretreatment steps need to go through are roughly as follows: tea-tree fruit—cold air drying—stripping—unshelled tea seed—roasting—hulling—cleaning—tea seed kernels. Three key factors correlated with the water content that affect the processing efficiency and the quality of the finished oil: (1) the harvested tea seeds with high water content are prone to mildew, which damages the quality of the finished oil; (2) unshelled tea seeds with high moisture content are hard to be hulled; (3) and seed kernels with low moisture content can reduce the oil yield. Therefore, a fast, accurate moisture measurement of tea-tree fruit without breaking its shell is urgent demanded in the processing of oil extraction. Therefore, a fast, accurate moisture measurement of tea-tree fruit without breaking its shell is urgent demanded in the processing of oil extraction.

Microwave sensing techniques offer a viable solution for evaluating the internal quality of food materials because microwaves can penetrate inside the materials [4,5,6,7,8]. When the microwave passes through a water-containing materials, the microwave energy loss caused by water is much greater than that caused by other dry substances, and the moisture content of substance significantly affect its dielectric constant [9]. Thus, the moisture content of the material can be determined indirectly by using the changes in key microwave features related to the dielectric constant (such as amplitude, phase angle, etc.) [10]. Because of non-contact with the measured material, fast response speed and good representativeness of measurement results, free-space transmission measurement methods are an ideal way to realize on-line measurement [11,12,13]. Compared with other indirect detection methods (near-infrared (NIR) method, resistance method, etc.) [14,15], the microwave space wave method can detect the overall moisture content of large quantities of accumulated materials. Moreover, the powerful microwave can penetrate tea seed kernels and be used to test the moisture content of the internal materials without breaking the shell [16,17,18].

Though multi-frequency scanning mode effectively can widen the moisture detection range for microwave technology [19,20], the high redundancy and multicollinearity among the multi-dimensional microwave frequency features limit detection efficiency. Therefore, the optimization of the microwave attenuations and phase-shifts is an essential task for the rapid moisture measurement [21,22]. In feature optimization, the final optimization results vary with the partitions of original feature set. To reduce the randomness introduced by feature set division and objectively optimize microwave detection frequency, a feature evaluation method that combines cross-validation and majority voting rules was proposed in this study, which can calculate the selection probability for each feature by their performance in multiple rounds of cross-validation.

In general, the main works in this study are: (1) a self-developed microwave moisture measuring system was introduced to determine the moisture content for unshelled tea seeds, (2) a feature optimization method with selective probability was designed to optimize the microwave attenuations and phase-shifts, (3) a moisture calibration function was constructed and validated to predict the kernel moisture from unshelled seed moisture.

## 2. Materials and Methods

### 2.1. Sample Preparation

**Figure 1:**
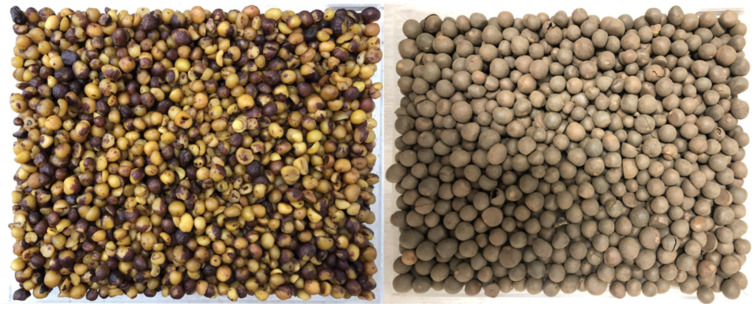
Tea seed kernels and unshelled tea seeds.

Green tea plant is a perennial evergreen tree, the green seed harvested in autumn (September–November in most regions) once a year, and the ideal moisture content for tea seeds at harvest is 20–30%. In the study, unshelled tea seeds were picked and dried in November 2022 (located in Jinhua, Zhejiang Province, China), and half of them were shelled and cleaned to obtain tea seed kernels. Both samples of unshelled tea seeds and shelled seed kernels were then sent to the laboratory in the Biosystems Engineering Department of Zhejiang University for the next preparation. Twelve batches of tea seed kernel samples, and 12 batches of unshelled tea seed samples at different moisture levels were prepared by misting them with modest amounts of distilled water. They were all sealed with a plastic bag and stored in a medical freezer (4 °C) for 72 h to equilibrate, and were thoroughly mixed to avoid uneven distribution of water. The wet basis moisture content of every batch of samples was determined with the oven-drying method [23]: the sample were repeatedly dried to constant weight in an oven at 105 °C, and its true moisture content was calculated according to the mass change of the sample before and after drying. Table 1 lists detailed physical characteristics of tea seed samples, and Figure 1 shows the tea seed kernel and unshelled tea seed samples.

### 2.2. Microwave Moisture Detection System

**Figure 2:**
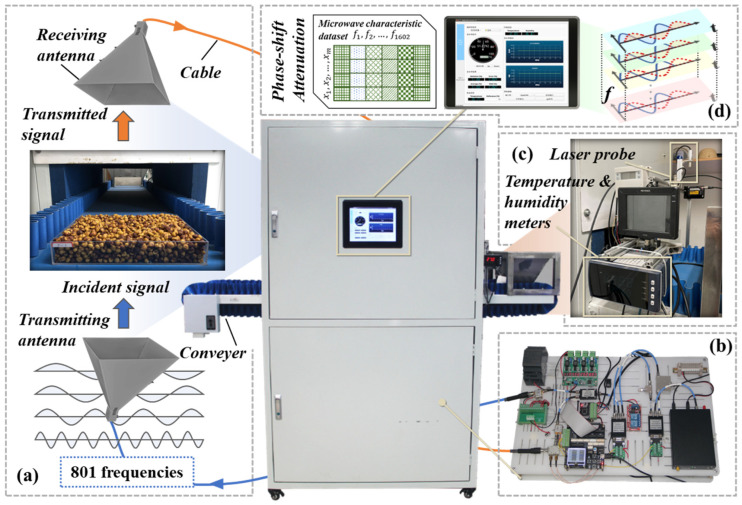
Structure diagram of the agricultural material moisture detection system based on multi-frequency microwave technology: (**a**) multi-frequency microwave measuring platform (MMP); (**b**) signal processing system (SPS); (**c**) external sensing module (ESM); (**d**) GUI of the human–computer interaction module (HIM).

A self-developed microwave moisture detection system based on multi-frequency sweep technology was taken to collect the microwave feature data of samples, which is mainly composed of four parts: multi-frequency microwave measuring platform (MMP), signal processing system (SPS), external sensing module (ESM), and human–computer interaction module (HIM) (Figure 2).

The signal processing system is the core part of the whole moisture detection system. It is mainly responsible for the transmission, reception, and analysis of sweep microwave signals, and finally the output of electrical signals containing microwave feature information (attenuation and phase shift).

**Figure 3:**
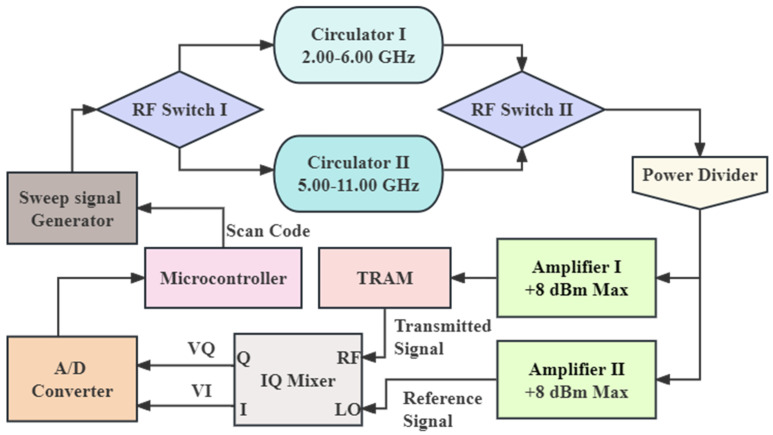
Microwave circuit diagram of signal processing system (TRAM, transmit–receive antenna module).

Figure 3 is a schematic diagram of the microwave circuit, in which main microwave devices applied are listed in Table 2. The transmission path of multi-frequency microwave signals is as follows. Firstly, the microwave signal source generates and transmits a sinusoidal signal at a specified frequency and power. The unidirectional transmission of the signal is achieved through the broadband isolator (the circulator with 2.00–6.00 GHz and the circulator with 5.00–11.00 GHz), which absorbs most of the reflected waves generated by the load to isolate its interference with the signal source. The working frequency bands of two circulators are 2.00 GHz–6.00 GHz and 5.00 GHz–11.00 GHz, respectively, which can be switched using radio frequency switches 1 and 2, respectively. Secondly, the microwave power was evenly distributed into two equal signals by the power splitter. One signal is modulated by a power amplifier as measuring signal, and converted into a spatial wave by the transmitting antenna, then the measuring signals passing through the material and captured by receiving antenna, and converted into input signal to RF port of IQ mixer. The other signal is modulated as the reference signal by the same power amplifier, and then input to LO port of IQ mixer. Finally, IQ mixer analyzes the two signals. If signals VI and VQ output by port Q and port I carry attenuation and phase shift information of microwave signal, this can be expressed as Equations (1)–(5) [24]:

(1) $$V^{I}=\frac{k}{4}V_{\mathrm{LO}}V_{\mathrm{RF}}\cos\phi_{\mathrm{LR}}$$

(2) $$V^{Q}=\frac{k}{4}V_{\mathrm{LO}}V_{\mathrm{RF}}\sin\phi_{\mathrm{LR}}$$

(3) $$P=10\log_{10}\left(\frac{1}{2R}\left({V^{I}}^{2}+{V^{Q}}^{2}\right)\cdot 10^{-3}\right)$$

(4) $$\phi_{\mathrm{LR}}=\tan^{-1}\left(\frac{V^{I}}{V^{Q}}\right)\cdot \frac{180}{\pi }$$

(5) $$∆\phi =\left(\phi_{\mathrm{no}\_\mathrm{load}}-\phi_{\mathrm{load}}\right)-360n$$

### 2.3. Multi-Frequency Microwave Scanning Measurement

The multi-frequency microwave scanning process of the samples are as follows: Firstly, the samples to be tested is evenly filled into a transparent open sample box (the bottom size is 300 × 240 mm, the height is the same as the material thickness which will be measured by the laser sensor IX-150 (KEYENCE, Osaka, Japan) and uploaded synchronously through the supporting host and communication module). Secondly, the material transmission device is started to send the samples into the microwave darkroom until the center point of the sample box is aligned with the central axis of the antenna receiving and sending group. Thirdly, the scanning range of microwave signal is set as 2.00–10.00 GHz (0.01 GHz interval), and the transmitting power is set to 14 dBm. Finally, the microwave signals (including 801 frequencies from 2.00 GHz to 10.00 GHz) are successively transmitted through the sample, and the microwave signals penetrating the sample were converted into electrical signals (such as amplitude attenuation, phase angle).

In the formal test, three no-load measurements were conducted initially (with an empty sample box) and the average value was taken as a reference. Then the samples to be measured were loaded for multi-frequency signal scanning measurement. The collected measurement values were subtracted from the reference values to get the real attenuation and phase shift values caused by the samples.

### 2.4. Data Processing

#### 2.4.1. Frequency Optimization with Selective Probability

**Figure 4:**
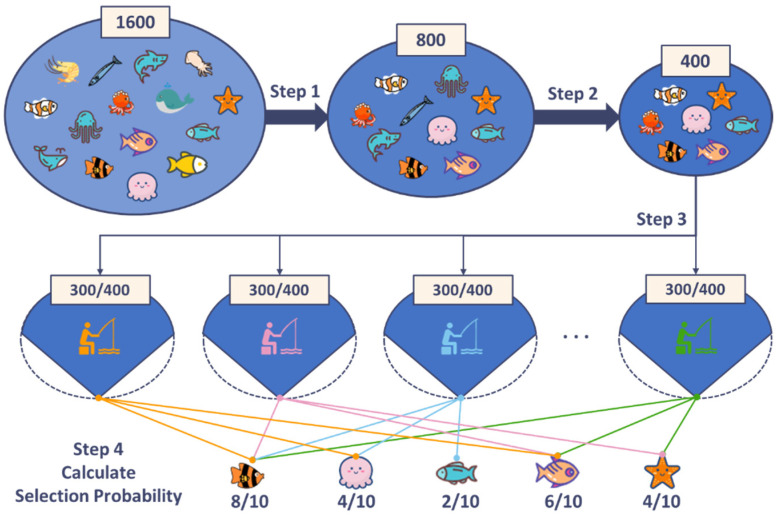
Diagram of the feature evaluation mechanism.

In frequency optimization, the final optimization results are often affected by differences in data set division. To reduce the randomness introduced by sample set division and objectively optimize microwave detection frequency, a frequency evaluation method combining cross-validation and majority voting rules was proposed. The key idea of the method was to record and evaluate the cumulative selection probability of each frequency after multiple rounds of cross-validation. the execution principle of the frequency evaluation mechanism is explained in Figure 4.

In the proposed feature selection, the setting of probability threshold is a subject deserving of study. Because when the probability threshold is too high (more than 0.6), the feature optimization of the multi-frequency microwave data is excessive, and the number of features retained is too small to meet the requirements of high-precision detection. When the probability threshold is too low (less than 0.3), over one hundred features can be selected, which will greatly prolong the scanning time of the multi-frequency microwave system, and it is not conducive to the rapid detection of the moisture content of the whole tea seed kernel. In our experiment, to balance the number of features and microwave scanning efficiency, the probability threshold was set to 0.4 through pre-testing.

#### 2.4.2. Modeling and Evaluation Methods

The multi-frequency microwave data set contains several samples, from which 70%, 15%, and 15% sample data were extracted to form the training set, verification set, and test set, respectively. Each dataset contains optimized microwave frequencies (divided from the original spectrum data according to the frequency subset) and sample thickness feature (fed back by the IX-150 sensor). The training set was taken to train the moisture prediction model, the validation set was taken to adjust the model parameters, and the test set was taken to test the prediction performance of model.

Artificial neural network (ANN) is a generic non-linear function approximator extensively used for pattern recognition and classification [25,26,27], which is a two-layer feed-forward network with sigmoid hidden neurons and linear output neurons. For the parameter optimization and quality evaluation of the ANN model, the input dataset is subdivided into three subsets, which are the training, validation, and test sets, respectively. The network is trained on the training set, and adjusted according to the fitting error [28]. The validation set is taken to measure the generalization ability of the network, the training progress will be halted if there is no longer improvement of the generalization. The test set is used to independently evaluate the fitting performance during and after training without any effect on the network.

#### 2.4.3. Software Environment

The experiments were carried out on an Intel Core-i5-10300H CPU with 16 GB DDR4 RAM (Intel Corp., Santa Clara, CA, USA) using the MATLAB R2020b implementation. The Statistics and Machine Learning Toolbox in MATLAB performed the algorithms with optimized parameters.

## 3. Results and Discussion

### 3.1. Tea Seed Kernels

#### 3.1.1. Analysis of Microwave Feature Spectrum

**Figure 5:**
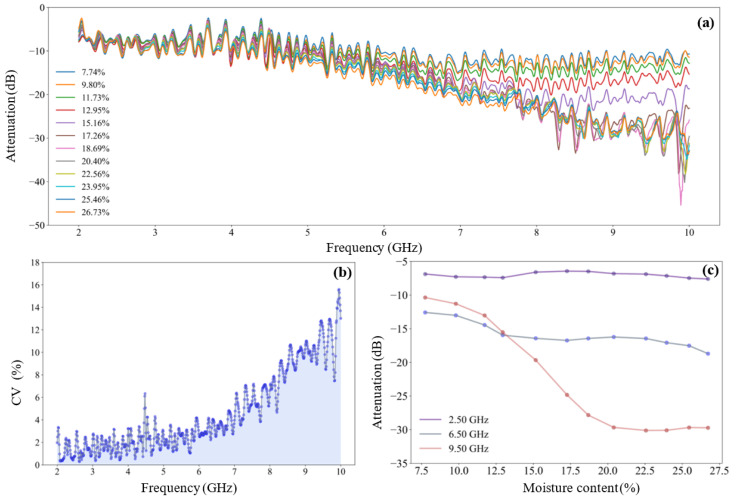
Attenuation spectrum of microwave signal of tea seed kernels on the different moisture levels: (**a**) Attenuation spectrum of sample on the different moisture levels; (**b**) coefficient of variation of attenuation values at different frequencies; (**c**) the variation curve of microwave signal attenuation with sample moisture content at 2.50 GHz, 6.50 GHz, and 9.50 GHz frequencies.

Figure 5 is the attenuation spectrum of microwave signal of tea seed kernels on the different moisture levels, where Figure 5a is the overview of the attenuation spectrum, and Figure 5b reveals the coefficient of variation of attenuation values at different frequencies. In the whole frequency band, the attenuation values of all samples are monotonically declining. With the increase of signal frequency, the divergence of attenuation spectrum of samples with different water content gradually increases. In the low and middle frequency band of 2.00–6.50 GHz, the overlap of spectral lines is serious, and the coefficient of variation is less than 3%; while in the middle and high frequency band of 6.50–10.00 GHz, the spectrum is gradually dispersed. The coefficient of variation gradually increases from 3% to 16% in this band.

For example, Figure 5c shows the variation of microwave attenuation with the moisture content of tea seed kernel samples at the 2.5 GHz, 6.50 GHz, and 9.50 GHz frequencies. It can be clearly seen that with the increase of moisture content, the attenuation value at 2.5 GHz fluctuates within a small range and the variation of the attenuation value at 6.50 GHz is only about 5 dB, indicating that the attenuation value at low and intermediate frequencies is less affected by the change of water content. For microwave signals at 9.50 GHz, the attenuation value varies greatly (about 20 dB) within the moisture content range of 7.74% to 20.40%, and the moisture content of sample is well differentiated. Within the moisture content range of 22.56% to 26.73%, the attenuation values change insensitive, which indicates that the difference of attenuation value caused by high water content samples at high frequency is not obvious.

**Figure 6:**
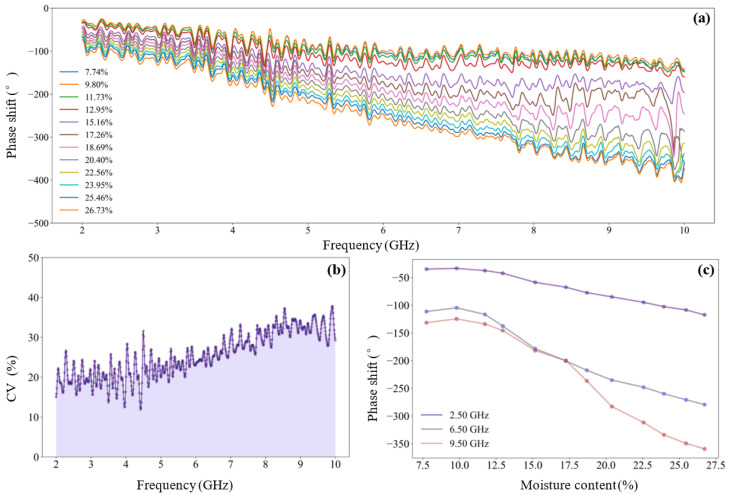
Phase shift spectrum of microwave signal of tea seed kernels on the different moisture levels: (**a**) Phase shift spectrum of sample on the different moisture levels; (**b**) coefficient of variation of attenuation values at different frequencies; (**c**) the variation curve of microwave signal phase shift with sample moisture content at 2.50 GHz, 6.50 GHz, and 9.50 GHz frequencies.

Figure 6 is the phase shift spectrum of microwave signal of tea seed kernels on the different moisture levels, where Figure 6a is the overview of the phase shift spectrum and Figure 6b reveals the coefficient of variation of phase shift values at different frequencies. As can be seen from Figure 6a, the phase shift value has a more regular monotone decline trend than the attenuation value, accompanied by small fluctuations, which better reflects the monotone variation relationship between the phase response of microwave signal and medium moisture content. In the whole frequency band, the dispersibility of the phase shift spectrum is better than that of the attenuation spectrum, and its coefficient of variation also increases from 20% to 35% with the increase of frequency (see Figure 6b). It indicates that the overall resolution of the phase shift feature to the water content of sample is better than that of the attenuation feature.

Similarly, Figure 6c plots the variation curve of microwave phase shift with the moisture content of tea seed kernels at the frequencies of 2.50 GHz, 6.50 GHz, and 9.50 GHz. Firstly, the variation amplitude of the phase shift value increases one by one at 2.50 GHz, 6.50 GHz, and 9.50 GHz (it is about 60°, 200°, and 240°, respectively), indicating that there is a certain difference in the ability of the signal to sense water at low, medium, and high frequencies. Secondly, the three phase shift curves all maintain a stable changing rate, indicating that the microwave phase shift with the same frequency is relatively consistent in the detection of the whole range of moisture content.

#### 3.1.2. Optimization of Microwave Frequency Features

**Figure 7:**
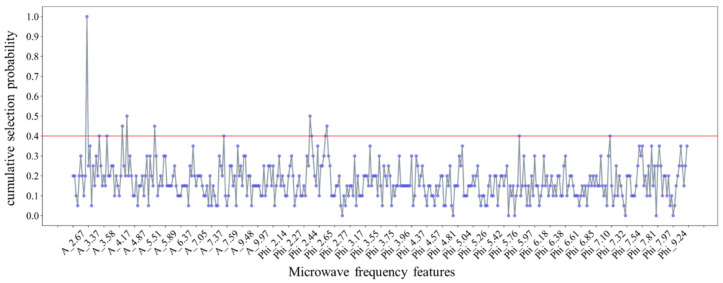
Cumulative selection probability of microwave frequency features of tea seed kernels.

The feature evaluation mechanism proposed in Section 2.4.1 was adopted to optimize the microwave frequency features for the detection of tea seed kernels, and the cumulative selection probability of all features was calculated (shown in Figure 7). The probability threshold was set as 0.4, and 13 preferred frequency features were obtained (including seven attenuations and six phase-shifts): [‘A_3.36’, ‘A_3.48’, ‘A_3.60’ and ‘A_4.32’, ‘A_4.37’, ‘A_5.54’, ‘A_7.55’, ‘Phi_2.57’, ‘Phi_2.58’, ‘Phi_2.70’, ‘Phi_2.72’, ‘Phi_5.97’, ‘Phi_7.30’].

#### 3.1.3. Moisture Prediction of Tea Seed Kernels

The ANN model based on the optimized multi-frequency microwave dataset was established for the moisture determination of tea seed kernels. Concretely, the moisture content of tea seed kernels (7.74–26.73%) was taken as the dependent variable, and the microwave features selected in Section 3.1.2 and the sample thickness feature were taken as the independent variables.

**Figure 8:**
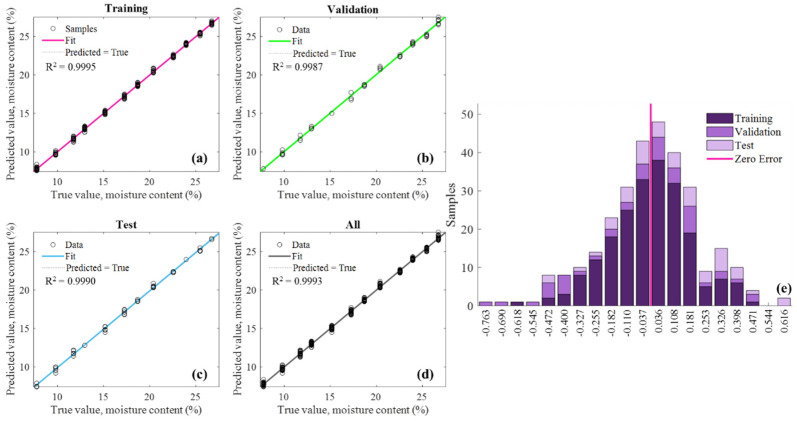
Prediction results for the moisture content of tea seed kernel samples based on the features selected from multi-frequency microwave dataset: (**a**) Training set (210 samples); (**b**) validation set (45 samples); (**c**) test set (45 samples); (**d**) all set (300 samples); (**e**) error histogram.

The prediction model was repeated 10 times with different random seed kernels, and all those regression results were shown in Table 3. It can be seen that the average regression scores of 10 independent runs on the validation and test sets were MSE_v_ = 0.213, RMSE_v_ = 0.371, $R_{v}^{2}$ = 0.998, and MSE_t_ = 0.164, RMSE_t_ = 0.375, $R_{t}^{2}$ = 0.998, respectively, which indicates that the model performed extremely well in predicting the moisture content of tea seed kernels based on multi-frequency data. Moreover, Figure 8 visualized the prediction results of the ANN model in one iteration. It could be seen from Figure 8a–d that all the determination coefficients of the prediction model based on the training, validation, and test data sets had exceeded 0.999. Meanwhile, all the scatters representing the tea seed kernels closely disturbed around the colored fit line, and the error histogram showed in Figure 8e demonstrated that the prediction errors of all samples complied with the normal distribution. The above results proved that the prediction model could achieve the high-precision detection of the moisture content of tea seed kernels and it exhibited the effectiveness of the rapid moisture determination of tea seed kernels based on the multi-frequency microwave technology. However, although the moisture measuring method has high precision, it still needs to break shell to acquire tea seed kernels, and the method fails to meet the requirement of non-destructive moisture detection for actual production. Therefore, the follow section carried out the study of moisture determination of unshelled tea seeds without breaking the seed shell.

### 3.2. Tea Seeds

#### 3.2.1. Analysis of Microwave Spectrum

**Figure 9:**
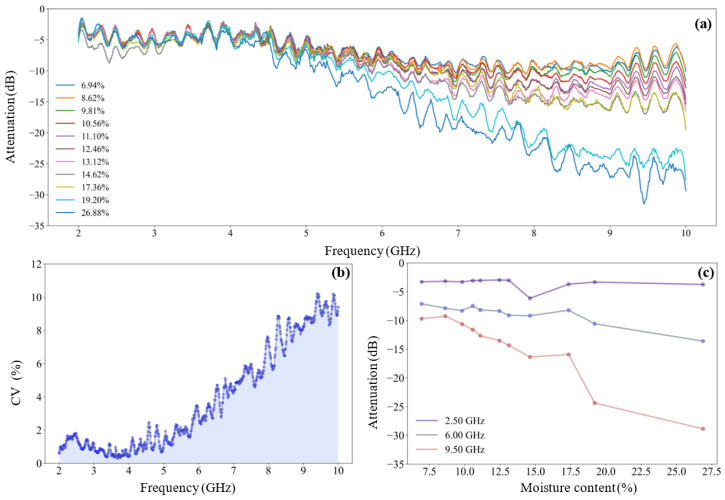
Attenuation spectrum of microwave signal of unshelled tea seeds on the different moisture levels: (**a**) Attenuation spectrum of sample on the different moisture levels; (**b**) coefficient of variation of attenuation values at different frequencies; (**c**) the variation curve of microwave signal attenuation with sample moisture content at 2.50 GHz, 6.00 GHz, and 9.50 GHz frequencies.

Figure 9 is the attenuation spectrum of microwave signal of tea seeds on the different moisture levels. Figure 9a shows that, the attenuation spectrum curves of all the tea seeds show a monotonically decreasing trend which is similar to the tea seed kernels, because the main components of the tea seed shell (such as cellulose, hemicellulose, and lignin) are weakly polar substances (relative dielectric constant 2.8–3.6). Among them, the component with the greatest influence on attenuation is still the moisture in the tea seeds (relative dielectric constant 78.36), so the microwave attenuation value gradually increases with the increase of frequency and the moisture content of the tea seeds. Based on the analysis of the coefficient of variation of Figure 9b, the two features of frequency and sample moisture content have no obvious effect on the enhancement of attenuation in the low frequency band of 2.00–5.00 GHz, the overlap degree of the spectrum curves is high and the coefficient of variation is stable at about 2%. However, in the middle and high frequency band of 5.00–10.00 GHz, the average slope of the spectrum curves gradually becomes larger and the sample with different moisture content gradually tend to be dispersed. The coefficient of variation gradually increases to about 10%, indicating that the attenuation values with the frequency band is more sensitive to the moisture content.

Similarly, the moisture sensing ability of the multi-frequency signal also shows some differences in different moisture content intervals. Figure 9c plots the variation curves of microwave attenuation with the moisture content at 2.50 GHz, 6.00 GHz, and 9.50 GHz frequencies to compare the differences of attenuation values at low, medium, and high frequencies. It can be seen that the higher the frequency, the greater the change rate of moisture content–attenuation curve, and the better the discrimination ability of microwave signals to moisture content. The ultimate goal of feature optimization for microwave frequency data is to select a group of attenuation features with the highest moisture content sensitivity.

**Figure 10:**
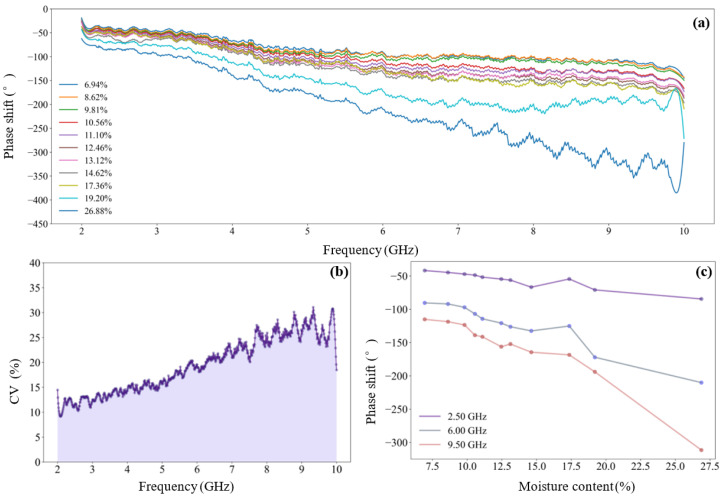
Phase shift spectrum of microwave signal of unshelled tea seeds on the different moisture levels: (**a**) Phase shift spectrum of sample on the different moisture levels; (**b**) coefficient of variation of attenuation values at different frequencies; (**c**) the variation curve of microwave signal phase shift with sample moisture content at 2.50 GHz, 6.00 GHz, and 9.50 GHz frequencies.

Figure 10 is the phase shift spectrum of microwave signal of tea seeds on the different moisture levels. As can be seen from Figure 10a, compared with the tea seed kernels, the phase-shift of tea seeds also show a more regular monotone downward trend, and the curves have small fluctuation. Combined with Figure 10b, it can be observed that the phase shift values exhibit obvious dispersion across the entire frequency spectrum, as evidenced by a gradual increase in coefficient of variation from 10% to 30%. Moreover, the phase shift values at the same frequency have higher coefficient of variation than the attenuation values, indicating that the resolution of phase shifts to unshelled seed samples with different water content is generally better than that of attenuations.

In order to further clarify the difference of phase values of tea seeds based on multi-microwave signal at low, medium, and high frequencies, Figure 10c plots the variation curve of microwave phase shift at 2.50 GHz, 6.00 GHz, and 9.50 GHz frequencies with different moisture content. It is clear that the higher the frequency, the greater the change rate of moisture content-phase shift curve. It can be seen from Figure 10c that when the moisture content of tea seeds increases from 7.5% to 27.5%, the phase shift ranges of microwave signals at 2.5 GHz, 6.0 GHz, and 9.5 GHz are 20°, 100°, and 200°, respectively. It is worth noting that the phase response at 2.50 GHz is not strong enough for the samples with water content ranging from 6.94% to 19.20%, and the resolution of the samples with different water content is poor. The change trend of phase shift of signals at 6.00 GHz and 9.50 GHz is consistent, but the curve does not decline monotonously in this interval. This indicates that the phase shift at medium and high frequencies are difficult to completely distinguish samples with different water content. Above analysis of the tea seeds shows that microwave attenuation and phase shift have certain differences in moisture content sensing.

#### 3.2.2. Optimization of Microwave Frequency Features

**Figure 11:**
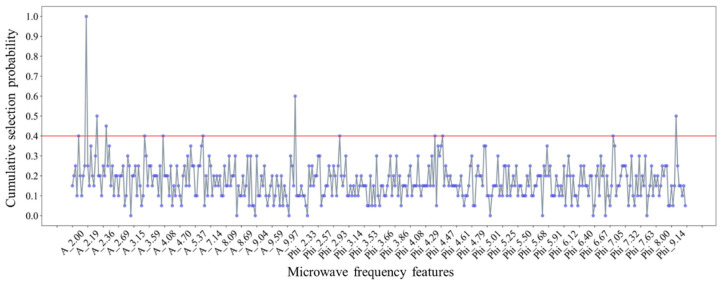
Cumulative selection probability of microwave frequency features of unshelled tea seeds.

The feature evaluation method proposed in Section 2.4.1 was adopted to optimize the microwave frequency features of tea seeds, and the selection probability of all features was calculated (shown in Figure 11). The probability threshold was set as 0.4, and 13 preferred frequency features were obtained: [‘A_3.36’, ‘A_3.48’, ‘A_3.60’, and ‘A_4.32’, ‘A_4.37’, ‘A_5.54’, ‘A_7.55’, ‘Phi_2.57’, ‘Phi_2.58’, ‘Phi_2.70’, ‘Phi_2.72’, ‘Phi_5.97’, ‘Phi_7.30’], including seven attenuations and six phase-shifts.

#### 3.2.3. Moisture Calibration Function of Tea Seeds

**Figure 12:**
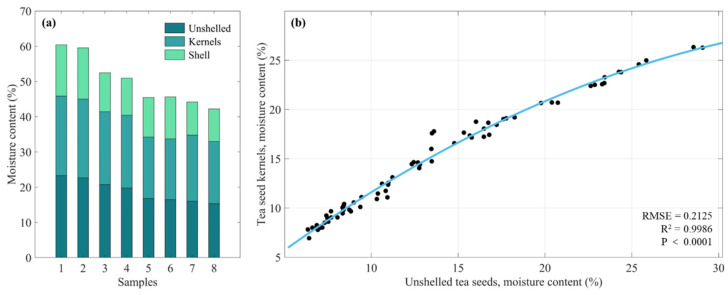
(**a**) Moisture contents of unshelled seeds, seed kernels and seed shells of tea; (**b**) variation of the moisture content of unshelled tea seed kernels with the moisture content of unshelled tea seeds.

To detect the moisture content of the seed kernel without breaking the shell, it is necessary to consider the influence of the moisture in the seed shell on the detection results. Figure 12a shows the moisture content of unshelled seeds, seed kernels, and seed shells in eight group samples. It can be seen that the shell has the lowest moisture content, the kernel has the highest moisture content, and the moisture content of unshelled seeds is between them.

In order to explore the correlation between the moisture content of tea seeds and tea seed kernels, 70 groups of tea seed samples with different moisture content were prepared. Taking polynomial to fit the moisture content of tea seeds (independent variable *y*) and tea seed kernels (dependent variable *x*), the fitting result is shown in Figure 12b. Overall, the fitting result is very good (RMSE = 0.2125%, R^2^ = 0.9986, *p* value < 0.0001), which illustrates a strong correlation in the moisture content of 5–30%. The fitting equation is shown in Equation (6):

(6) $$y=-0.017x^{2}+1.431x-1.019$$

where three coefficients (they are −0.017, 1.431 and −1.019, respectively) reveal the difference between the moisture content of tea seeds and tea seed kernel. Therefore, this equation can be used as a moisture calibration function to realize the kernel moisture detection of unshelled tea seeds. The moisture content of unshelled tea seeds is firstly predicted according to the multi-frequency microwave data, then the moisture calibration function is used to calibrate the predicted value, and finally outputs the predicted moisture content of seed kernels.

**Figure 13:**
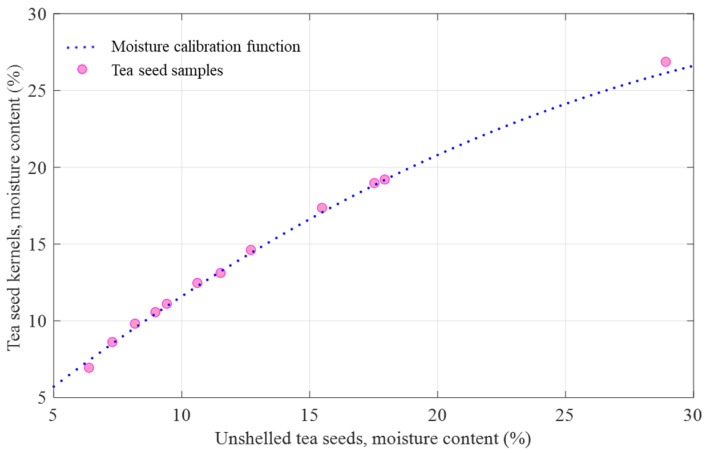
Validation of the moisture calibration function.

To verify the feasibility and accuracy of the moisture calibration function, through this function, the moisture content of tea seeds is converted into that of tea seed kernels. Both the moisture calibration curve and the moisture content data of 12 batches of tea seed samples (every tea seed sample was measured in the form of tea seed and tea seed kernel) were visualized in figure. The moisture content of tea seeds was taken as the *x*-axis, and the moisture content of tea seed kernels was taken as the *y*-axis, as shown in Figure 13. From Figure 13, all the scatters are regularly distributed along the moisture calibration curve, and no scatter significantly deviates from it. Therefore, the verification results showed that the moisture calibration function accurately mapped the moisture content of tea seeds to that of tea seed kernels, which could be used for the moisture calibration.

#### 3.2.4. Moisture Prediction of Tea Seeds

The ANN model of moisture content of tea seeds was established using optimized multi-frequency microwave dataset, and the ANN prediction results would be compared with those using the moisture calibration function. The moisture content of tea seeds (6.94–26.88%) was taken as the dependent variable, and the microwave features selected in Section 3.2.2 and the sample thickness features were taken as the independent variables.

**Figure 14:**
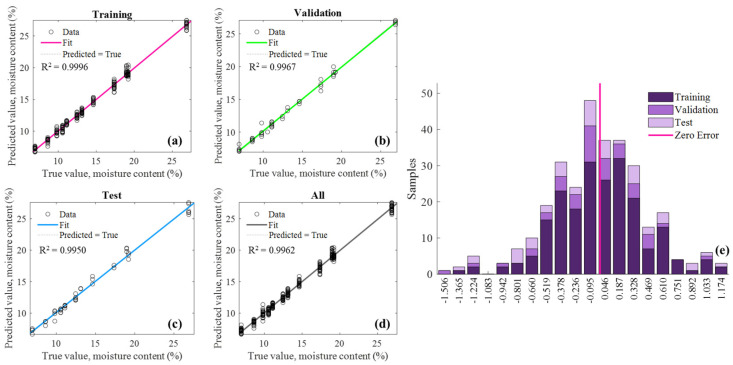
Prediction results for the unshelled kernel moisture content of unshelled tea seed samples based on the features selected from multi-frequency microwave dataset: (**a**) Training set (210 samples); (**b**) validation set (45 samples); (**c**) test set (45 samples); (**d**) whole set (300 samples); (**e**) error histogram.

The prediction model was independently repeated 10 times, and the regression results were shown in Table 4. The model exhibited good performance in predicting the moisture content of tea seed kernels, its average regression scores of 10 independent runs on the validation and test sets were MSE_v_ = 0.558, RMSE_v_ = 0.698, $R_{v}^{2}$ = 0.990, and MSE_t_ = 0.868, RMSE_t_ = 0.861, $R_{t}^{2}$ = 0.988, respectively. Figure 14 visualized the prediction results of the ANN model in one iteration, including its fitting results and prediction error for samples in the training, validation, test, and whole sets. It can be seen from Figure 14a–d that the determination coefficients of the prediction model on the training, validation, test, and all sets were 0.999, 0.997, 0.995, and 0.996, respectively, and Figure 14e showed that the prediction errors of all samples basically fit the normal distribution. The above results proved that the presence of seed shells had little effect on the moisture prediction of unshelled kernels, and it was feasible to directly determine the moisture content of seed kernels without breaking shell. which could be because the constructed prediction model had successfully learnt the proportional relationship between the moisture content of unshelled tea seeds and seed kernels.

**Figure 15:**
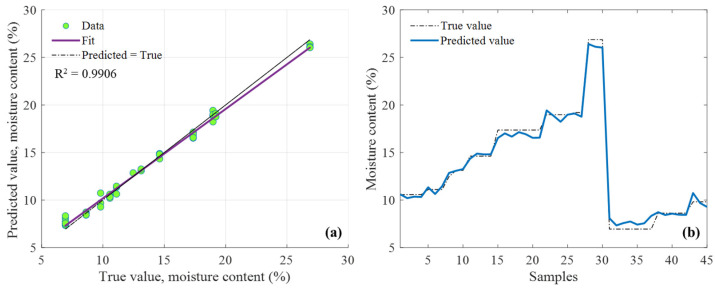
Prediction results for the unshelled kernel moisture content of unshelled tea seed samples based on the predicted value of unshelled tea seed moisture content with the moisture calibration function: (**a**) Correlation analysis between of the predicted and true values of test samples; (**b**) comparison of the predicted and true values of test samples.

As shown in Figure 15, the kernel moisture content of unshelled tea seeds was detected based on the multi-frequency dataset with the moisture calibration function. Firstly, the ANN prediction model for the moisture content of unshelled tea seeds was constructed, the true value of the moisture content of unshelled tea seeds (6.38–28.92%) was taken as the dependent variable, and the microwave features selected in Section 3.2.2 and the sample thickness feature were merged as the independent variables. Secondly, the predicted values of moisture content of unshelled tea seeds were calibrated by the moisture calibration function, through which the predicted moisture contents of unshelled tea seeds were mapped to those of its unshelled kernels.

The prediction model was independently repeated 10 times, and the regression results were shown in Table 5. The ANN model had excellent performance in predicting the moisture content of unshelled tea seeds (shown in the “Uncalibrated” columns in Table 5), whose average regression scores of 10 independent runs on the test set were MSE_t_ = 0.372, RMSE_t_ = 0.591, $R_{t}^{2}$ = 0.991, respectively. In summary, the prediction accuracy after calibration decreased slightly (from 0.996 to 0.989). This loss of accuracy may be caused by the introduction of moisture calibration function. However, the prediction performance of the ANN model built with moisture calibration function (0.988 ± 0.011) was close to the ANN model built without moisture the calibration function (0.989 ± 0.003) discussed in Section 3.2.3, and Figure 15 visualized the moisture prediction results for the unshelled tea seed kernel based on the predicted moisture content value of unshelled tea seed with the moisture calibration function. This method innovatively represents the influence of seed shell on moisture detection by a moisture calibration function which reduced the influence of seed shell to the kernel moisture content detection, and it shows promising application potential in the non-destructive unshelled kernel moisture detection of other agricultural materials with shells.

## 4. Conclusions

In this study, the moisture prediction results validated the feasibility of the non-destructive detection of unshelled tea seed moisture contents using a multi-frequency microwave system. The proposed feature optimization method significantly enhanced detection efficiency by selecting the optimal microwave frequency feature, and the constructed moisture calibration function demonstrated a strong correlation between the moisture content of tea seeds kernels and unshelled tea seed kernels, ensuring the accuracy of the moisture prediction. By establishing an ANN-moisture correction function model and conducting repeated experiments for verification, it was confirmed that the moisture content of tea seed kernels could be predicted by optimizing the multi-frequency microwave features of unshelled tea seeds. Therefore, the results of this study suggest that the microwave device can realize the non-destructive testing of the moisture content of the internal materials without breaking the shell.

## Figures and Tables

**Table 1:** Physical characteristics of tea seed samples.

Material	Diameter (mm)	Moisture Content (%)	Batch Number
Tea seed kernels	8–10	7.74–26.73	12
Unshelled tea seeds	12–15	6.94–26.88	12

**Table 2:** Detailed information about main microwave devices in signal processing system.

Device	Model	Manufacturer
Signal generator	SG24000H	DS INSTRUMENTS Inc., Gardnerville, NV, USA
Circulator	D3C2060/DMC5011	DiTom Microwave Inc., Fresno, CA, USA
RF switch	R12-SF12T18-D	Talent Microwave Inc., Suzhou, CHN
Power divider	RS2W20180-S	Talent Microwave Inc., Suzhou, CHN
Amplifier	ZX60-123LN-S+	Mini-Circuits Inc., Brooklyn, NY, USA
IQ mixer	MLIQ0218L	Marki Microwave Inc., Morgan Hill, CA, USA

**Table 3:** MSE, RMSE, and R^2^ (results of 10 independent runs) of the moisture prediction model of tea seed kernels on the validation (v) and test (t) sets.

Iterations	MSE_v_	MSE_t_	RMSE_v_	RMSE_t_	$R_{v}^{2}$	$R_{t}^{2}$
1	0.047	0.089	0.217	0.298	0.999	0.999
2	0.072	0.063	0.268	0.251	0.999	0.999
3	0.050	0.104	0.223	0.322	0.999	0.998
4	0.104	0.128	0.322	0.357	0.998	0.998
5	0.082	0.076	0.287	0.276	0.999	0.999
6	0.078	0.374	0.280	0.611	0.999	0.995
7	0.102	0.093	0.319	0.305	0.999	0.999
8	1.409	0.536	1.187	0.732	0.988	0.994
9	0.087	0.103	0.295	0.321	0.999	0.998
10	0.100	0.074	0.316	0.271	0.999	0.999
Average	0.213	0.164	0.371	0.375	0.998	0.998
Std.	0.421	0.159	0.289	0.162	0.004	0.002

**Table 4:** MSE, RMSE, and R^2^ (results of 10 independent runs) of the moisture prediction model of tea seeds on the validation (v) and test (t) sets.

Iterations	MSE_v_	MSE_t_	RMSE_v_	RMSE_t_	$R_{v}^{2}$	$R_{t}^{2}$
1	0.866	1.365	0.931	1.168	0.988	0.982
2	0.200	0.455	0.447	0.675	0.998	0.993
3	1.617	0.698	1.272	0.836	0.965	0.989
4	0.574	0.348	0.757	0.590	0.986	0.995
5	0.399	0.422	0.632	0.649	0.994	0.993
6	0.184	3.108	0.429	1.763	0.997	0.960
7	0.168	0.938	0.410	0.969	0.997	0.985
8	0.497	0.254	0.705	0.504	0.992	0.995
9	0.847	0.718	0.921	0.847	0.985	0.992
10	0.225	0.369	0.475	0.608	0.997	0.995
Average	0.558	0.868	0.698	0.861	0.990	0.988
Std.	0.455	0.855	0.281	0.375	0.010	0.011

**Table 5:** MSE, RMSE, and R^2^ (results of 10 independent runs) of the moisture prediction model on the test (t) sets.

Iterations	MSE_t_		RMSE_t_		$R_{t}^{2}$	
	Uncalibrated	Calibrated	Uncalibrated	Calibrated	Uncalibrated	Calibrated
1	0.144	0.280	0.380	0.529	0.998	0.991
2	0.270	0.267	0.519	0.516	0.995	0.988
3	0.429	0.353	0.655	0.594	0.996	0.991
4	0.868	0.495	0.932	0.704	0.994	0.982
5	0.334	0.376	0.578	0.613	0.995	0.985
6	0.565	0.364	0.752	0.603	0.992	0.987
7	0.186	0.198	0.431	0.445	0.997	0.991
8	0.253	0.234	0.503	0.484	0.996	0.990
9	0.385	0.308	0.620	0.555	0.996	0.991
10	0.288	0.270	0.537	0.520	0.998	0.992
Average	0.372	0.315	0.591	0.556	0.996	0.989
Std.	0.213	0.085	0.161	0.074	0.002	0.003

## Data Availability

The original contributions presented in this study are included in the article. Further inquiries can be directed to the corresponding author.
